# Long-term results of a phase II study of synchronous chemoradiotherapy in advanced muscle invasive bladder cancer

**DOI:** 10.1038/sj.bjc.6601852

**Published:** 2004-05-04

**Authors:** S A Hussain, D D Stocken, D R Peake, J G Glaholm, A Zarkar, D M A Wallace, N D James

**Affiliations:** 1 Cancer Research UK Institute for Cancer Studies, University of Birmingham, Birmingham B15 2TT, UK; 2 Queen Elizabeth Hospital, Edgbaston, Birmingham, B15 2TH, UK

## Abstract

We conducted a phase I/II study investigating synchronous chemoradiotherapy with mitomycin C and infusional 5-fluorouracil (5-FU) in muscle invasive bladder cancer. Early dose escalation results were previously published. We report the long-term toxicity and efficacy results with the optimised regimen. Patients with muscle invasive bladder cancer with glomerular filtration rate >25 ml min^−1^ were eligible. Mitomycin (12 mg m^−2^ on day 1 only) and infusional 5-FU (500 mg m^−2^ day^−1^) for 5 days were administered during weeks 1 and 4 of radiotherapy of 55 Gy in 20 fractions. A total of 41 patients were enrolled, median age was 68 years, 33 were male and eight female patients. Out of the 41 patients, 20 (49%) had hydronephrosis at presentation and 25 (62%) had T3b or T4 disease. Four patients experienced Grade III thrombocytopenia and three patients had Grade III neutropenia. There were no episodes of febrile neutropenia. Four patients experienced Grade III diarrhoea and 1 Grade III urgency and dysuria. Six patients did not undergo cystoscopic evaluation due to early metastatic spread although there was no clinical suggestion of bladder failure. In all, out of 35 evaluable patients, 25 (71%) had macroscopic complete response at 3-month cystoscopy, and biopsy confirmed in 24 out of 25. A total of 16 (39%) patients remain alive with a median follow-up of 50.7 (range 23.5–68.8) months, 14 with a functioning bladder with no reported long-term treatment-related bladder or bowel toxicity. Five out of 41 patients have undergone salvage cystectomy: two for persistent CIS, two T1 and one muscle invasive recurrence. Four patients have received intravesical chemotherapy, of whom two remain alive with a functioning bladder. Overall 12-, 24- and 60-month (m) survival rates were 68, 49 and 36%. Local and distant progression free rates were 82 and 86% at 12-m and 79 and 75% at 24-m. Organ preservation using multimodality therapy is feasible and safe, even in patients with poor renal reserve, and does not compromise salvage therapies. A national phase III trial BC2001 (www.bc2001.org.uk) exploring the effects of synchronous chemoradiotherapy with this regimen is currently recruiting.

The optimum management strategy for control of locally advanced bladder cancer remains to be determined. Surgical removal of the bladder may attain local control but 20-30% of patients may develop a local relapse (Hall and Dinney, 1996, Smith *et al*, 1997) and all will need either reconstructive bladder surgery or an ileal diversion. Improved surgical techniques have improved quality of life after cystectomy; however, even continent urinary diversion with orthotopic lower urinary tract reconstruction cannot substitute for the patient's original bladder. Radical radiotherapy is commonly used as an alternative to surgery especially in the UK. For every patient undergoing surgery in the UK there are two patients receiving radiation treatment (BAUS 2000). This approach suffers from a relatively high rate of incomplete response or local recurrence (up to 50%) with salvage cystectomy being used for failures (Parker and Huddart, 1996; Cooke *et al*, 2000).

## ROLE OF SYNCHRONOUS CHEMORADIOTHERAPY

There are indications from other primary sites that synchronous chemoradiotherapy may produce local control with or without survival advantages (Chan *et al*, 1993; Pritchard and Anthony, 1996; UKCCCR working party, 1996; Al-Sarraf *et al*, 1997; Haffty *et al*, 1997). A variety of phase II studies have investigated the efficacy and toxicity of this approach in bladder cancer generally with encouraging results (Hussain and James, 2002). Rödel *et al* (2002a) recently described a large experience with organ-sparing treatment of invasive bladder cancer, documenting long-term outcome of 415 patients treated over a 20-year time period showing promising results. Zietman *et al* (1993) and Shipley *et al* (1985, 2002) from Massachusetts General Hospital and Sauer *et al* (1998) from Erlangen have reported promising results with organ preservation strategies over the last decade that offered hope and an option for bladder preservation in a significant proportion of patients who currently undergo cystectomy.

## EVIDENCE FOR ORGAN PRESERVATION

We have previously reported results from our single centre phase I/II study investigating the role of synchronous chemoradiotherapy using mitomycin C and infusional 5-flourouracil (5-FU) in muscle invasive bladder cancer (Hussain *et al*, 2001). We now report the final results of long-term toxicity and efficacy of patients receiving the optimised regimen of 5 days 5-FU infusion with mitomycin C. Currently, the only randomised evidence for organ preservation in bladder cancer comes from a small trial by the national cancer institute of Canada (Coppin *et al*, 1996). This study randomised 99 patients to receive cisplatin 100 mg m^−2^ with radiotherapy or radiotherapy alone at a dose of 40 Gy followed by elective cystectomy or further radiotherapy. The chemoradiotherapy arm showed statistically nonsignificant improvements in complete response rate to radiotherapy. Significant differences were seen in the pattern of relapse. The use of cisplatin in bladder cancer is limited by its renal toxicity as significant proportions of patients have impaired renal function with hydronephrosis and additionally administration may require in-patient stay and hydration. We evaluated a combination of 5-FU, mitomycin C and radiotherapy in a phase I dose escalation study using a similar schedule adapted from that used in UKCCCR anal carcinoma study (UKCCCR working party, 1996). 5-Flourouracil is systemically active in bladder cancer (Carter and Wasserman, 1975; Logothetis *et al*, 1991; Huan *et al*, 1995). Mitomycin C is of value as a radiosensitiser (UKCCCR working party, 1996). Low-dose 5-FU and mitomycin C were tested in a small phase II study of predominantly T3/T4 patients and achieved a 74% clinical complete response (CR) rate and long-term survival of 54% (Rotman *et al*, 1990). The radiation treatment consisted of 4000–4500 rad over 4.5–5 weeks to pelvis and 2000–2500 rad boost.

## PATIENTS AND METHODS

Patients with histologically confirmed muscle invasive primary transitional cell carcinoma of the bladder (T2-4a N0/NX M0) were eligible for the study. Other entry criteria included ECOG performance status 0–2, written informed consent, no concomitant or previous malignancy likely to interfere with the protocol treatment or assessment, leucocytes >4 × 10^9^ l^−1^, platelets >100 × 10^9^ l^−1^, glomerular filtration rate (GFR), calculated by Cockcroft formula, >25 ml min^−1^, AST, ALP <1.5 × the upper limit of normal range. Prior to therapy, all patients underwent physical examination, haematological, renal and biochemical profile examination, CT scan of abdomen and pelvis, chest X-ray and examination under anaesthetic plus cystoscopic resection of tumour and biopsy. The TNM classification (1997) was used for staging purposes.

### Study design

This study had Local Research Ethics Approval and started enrolling patients in June 1998. The study was a combined phase I/II study. The early dose escalation part of this study has previously been reported (Hussain *et al*, 2001). The primary end point of the phase II portion of the study was pathological response in the bladder at 3 months with secondary end points of toxicity, time to progression and overall survival. Toxicity was assessed using the NCI grading system.

### Treatment

Treatment comprised chemotherapy and radiotherapy synchronously, with chemotherapy starting 1 h earlier than the commencement of radiotherapy on day 1 of the start of treatment.

### Radiotherapy

All patients were planned with the bladder empty to keep the clinical target volume as small as possible. The planning target volume (according to the ICRU 50 conventions) encompassed the normal bladder with a margin of 1.5 cm, and assessable tumour plus a margin of 2 cm. The anterior margin was also reduced to 1.5 cm where a larger margin would lie within the os pubis. Treatment was given to a single planning target volume. The patients were catheterised and 30 ml contrast medium introduced. Initial localisation films were taken on the simulator and fiduciary marks made before transfer to the CT scanner. CT images were transferred electronically to the radiotherapy planning system for mark-up. All patients were planned using computerised tomographic techniques on the central slice of the planning target volume. Typical field arrangements were an anterior open field and two wedged oblique posterolateral fields. Radiotherapy dose was 55 Gy in 20 fractions over 4 weeks, prescribed at the field intersection with less than ±5% variation across the planning target volume. Changes in fractionation due to machine breakdown, etc., were handled according to the recommendations issued by the Royal College of Radiologists on treatment delays: the preferred options being transfer to another machine or twice daily treatment with a 6 h gap thereby keeping the total duration of radiotherapy constant (www.rcr.ac.uk).

### Chemotherapy

The chemotherapy comprised single dose of mitomycin C and 5-FU as a continuous infusion. Concurrent chemotherapy with 5-FU 500 mg m^−2^ 24 h^−1^ was given during fractions 1–5 and 16–20 of radiotherapy treatment (total 10 days therapy). Mitomycin- C was given at a dose of 12 mg m^−2^ as a single bolus on day 1 of radiotherapy treatment. Chemotherapy was planned in an outpatient setting via a peripherally inserted central catheter (PICC) line.

### Response Assessment

All patients were scheduled for rigid check cystoscopy with tumour bed biopsy at 3 months post-treatment. A post-treatment CT scan was performed prior to cystoscopy to check for local or distant recurrence. Patients with metastatic spread did not undergo check cystoscopy, although local pelvic spread could be assessed on the scan. Subsequent cystoscopies were carried out at 6 months, then 6 monthly, with biopsy only if clinically indicated.

### Statistical considerations

All patients receiving treatment were included in the toxicity and survival analysis. Data were analysed when all alive patients had been followed for a minimum of 2 years (November, 2003). Response rates and toxicity data were analysed with simple descriptive statistics. Survival was calculated from date of diagnosis to date of death for any cause, or censor date for alive patients. Time to progression analysis censored patients at the time of death if they died without clinical evidence of progression. Survival and progression free estimates were calculated according to the method of Kaplan and Meier (1958).

## RESULTS

### Patient characteristics

A total of 41 patients entered the trial from March 1998 to August 2001 of whom 20 were in the phase I dose escalation portion of the study. Median age was 68 (range 58–79) years, with 33 male and eight female patients (Table 1

**Table 1:** Patient characteristics

	**Patients (%) *N*=41**	
Age (years)		
Median	68	
Range	58–77	
Sex		
Male	33	80%
Female	08	20%
Stage		
T2	10	24%
T3a	06	14%
T3b	14	34%
T4	11	28%
Grade		
2	10	24%
3	31	76%
Hydronephrosis		
Yes	21	51%
No	20	49%
GFR		
<50 ml min^−1^	17	32%
Performance status		
0	25	61%
1	11	27%
2	5	12%
Completing planned treatment		
Radiotherapy	41	100%
Chemotherapy	35	85%

).

### Toxicity

Toxicity was mild to moderate. A total of 35 patients (85%) received the full planned dose of 10 days of concurrent chemotherapy and all patients received the planned radiotherapy (Table 1). One patient declined to receive the second infusion because of grade 2 nausea, two patients received a 25% dose reduction during the second infusion due to GI toxicity and in two cases the second infusion was interrupted after 3 days, one due to grade 3 thrombocytopenia and one due to venous access complications. In one case, the second infusion was not instituted because of Grade III thrombocytopenia as per the protocol (Table 2

**Table 2:** Toxicity (maximum toxicity recorded)

**Toxicity**	**Patients (%) *N*=41**
Diarrhoea	
Grade 1	16 (39%)
Grade 2	13 (32%)
Grade 3	4 (10%)
Cystitis dysuria/urgency	
Grade 1	15 (36%)
Grade 2	7 (17%)
Grade 3	1 (2%)
Thrombocytopenia	
Grade 1	21 (51%)
Grade 2	7 (17%)
Grade 3	4 (10%)
Neutropenia	
Grade 1	20 (49%)
Grade 2	9 (22%)
Grade 3	3 (7%)

).

#### Haematological toxicity

The treatment was well tolerated with little in the way of haematological toxicity. On this regimen, four patients suffered from Grade III thrombocytopenia and three patients had Grade III neutropenia. There was no grade IV haematological toxicity and no episode of febrile neutropenia or neutropenic sepsis.

#### Nonhaematological toxicity

Cystitis and diarrhoea were the main nonhaematological toxicity encountered during the course of synchronous chemoradiation treatment. The treatment was well tolerated with four patients encountering Grade III diarrhoea and one patient experiencing Grade III urgency and dysuria.

### Chemotherapy in an outpatient setting

Chemotherapy was given in an outpatient setting via a PICC line in 33 patients, four patients opted for outpatient therapy but required in-patient treatment because of poor venous access and four patients opted for in-patient treatment.

### Response to therapy

**Figure 1:**
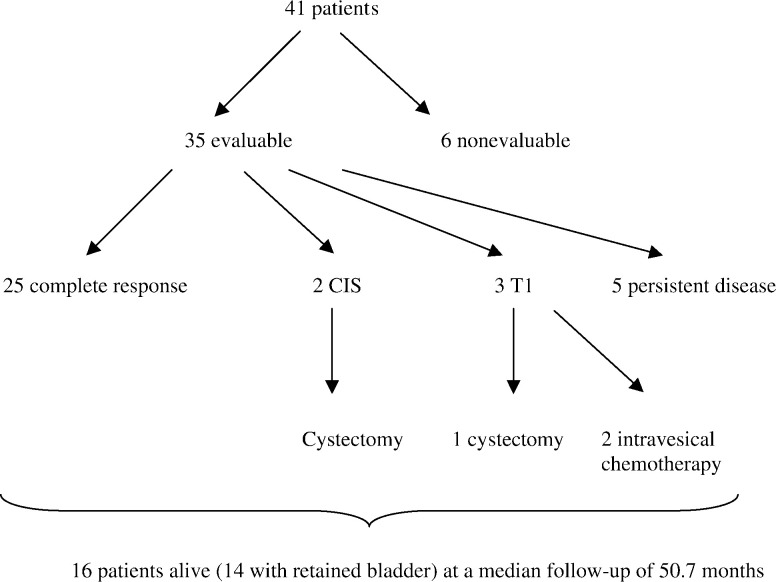
Response to therapy.

Of the 41 patients, 35 were eligible for local response assessment. Six of the 41 patients did not undergo cystoscopic evaluation due to early metastatic spread although there was no clinical suggestion of bladder failure. Of the 35 patients, 25 (71%) had macroscopic complete response at 3-month cystoscopy, confirmed by tumour bed biopsy in 24 out of 25 (intention to treat complete response rate 25 out of 41 (61%)). Two patients had residual carcinoma *in situ*, and one had T1 disease at cystoscopy with no muscle invasion and they underwent salvage cystectomy. Two patients with residual superficial carcinoma were treated with intravesical chemotherapy. The remaining five had persistent muscle invasive disease (Figure 1

). At a median follow-up of 51 months, 16 patients remain alive, 14 of them with functioning bladder. Of these sixteen alive patients, 12 have no signs of local progression, two relapsed with superficial disease and had intravesical chemotherapy, and two underwent salvage cystectomy for invasive recurrence 36 and 48 months post-treatment, respectively. In total, five out of 41 patients underwent salvage cystectomy. Seven patients with complete response in the bladder went on to develop distant metastases: one in the para-aortic lymph nodes that was treated successfully with CMV chemotherapy and local irradiation (this patient's bladder has remained free of disease throughout); in the second case, also with a durable local complete response, the patient developed brain metastases and died. A further five patients developed visceral metastasis and were treated with chemotherapy. Patterns of relapse are detailed in Tables 3

**Table 3:** Survival

	**Local progression**		**Distant progression**	
	**No**	**Yes**	**No**	**Yes**
Status				
Alive	12	4	15	1
Dead	12	7	11	9
Total	24	11	26	10

and 4

**Table 4:** Pattern of relapse

	**DP**		
	**No**	**Yes**	**Total**
LP			
No	17	7	24
Yes	9	2	11
Total	26	9	35

.

### Survival and time to progression analyses

**Figure 2:**
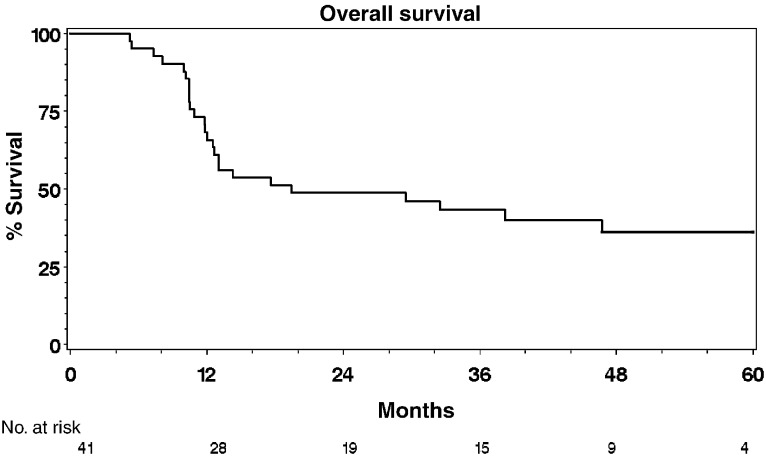
Overall survival.

**Figure 3:**
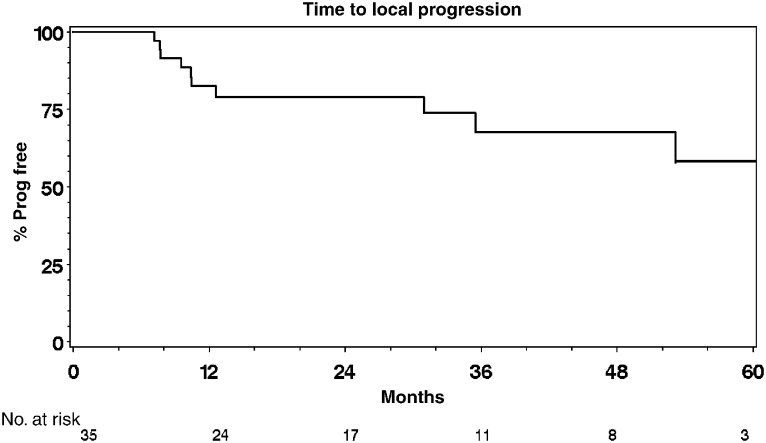
Time to local progression.

Of the 41 patients, 25 had died by the time of analysis with a median follow-up of 50.7 months (range 23.5–68.8 months) for the 16 alive patients. Overall, 12-, 24- and 60-month survival rates were 68% (95% CI: 54%–83%), 49% (CI: 33%–64%) and 36% (CI: 20%–52%) (Figure 2

). Of the 25 deaths, 15 were bladder cancer specific. The 12- and 24-month cause-specific survival was 77% (95% CI: 64–90%) and 68% (95% CI: 53–83%), respectively. Six patients were not evaluable for local progression and five were not evaluable for distant progression because of early death or distant progression, without any symptoms of local relapse or progression and did not undergo cystoscopy for local control assessment. Of the 35 patients, 11 had local progression and 10 of 36 patients had distant progression confirmed at the time of analysis. In all, 12 and 11 patients died without local or distant progression, respectively. These patients were censored and included in the analysis being at risk of progression up to their death (Tables 3 and 4). Local and distant progression free rates were 82% (CI: 70–95%) and 86% (CI: 75–97%) at 12 months and 79% (CI: 65–93%) and 75% (CI: 60–90%) at 24 months, respectively (Figures 3

and 4

**Figure 4:**
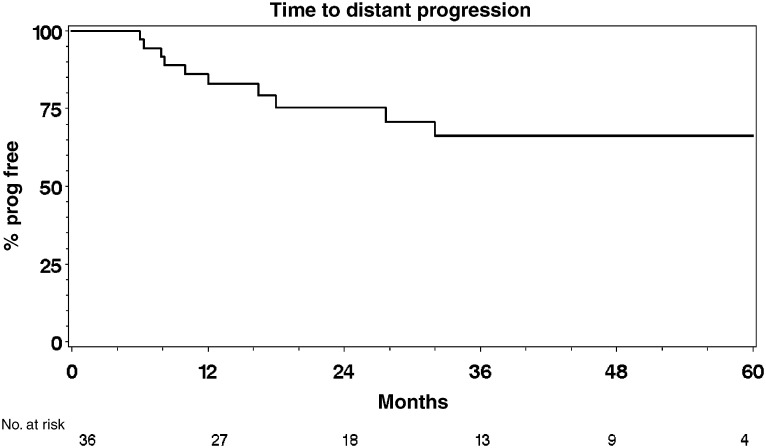
Time to distant progression.

).

### Renal function

**Figure 5:**
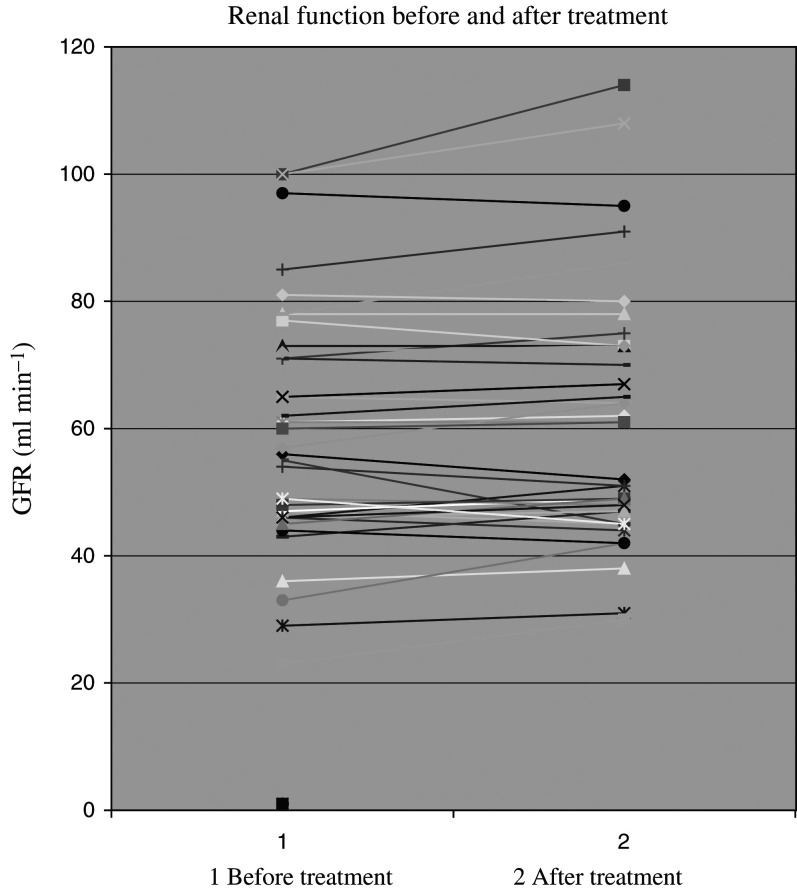
Effect of treatment on renal function.

The renal functions remained stable or improved while on treatment. No patient suffered a clinically significant drop in renal function as shown in Figure 5

.

## DISCUSSION

The initial phase I study showed that the addition of infusional 5-FU plus mitomycin C to full-dose radical radiotherapy is safe and feasible in patients with locally advanced bladder cancer (Hussain *et al*, 2001). Dose escalation from 5 days to 7 days, however, resulted in increased toxicity and more frequent dose modifications in both chemotherapy and radiotherapy particularly in the second half of the treatment schedule. Treatment in most cases was completed as an outpatient using an infusion pump and PICC line avoiding significant use of in-patient resources and increasing patient convenience. The 5-day infusion schedule was selected as the basis for the phase II study and this trial continued recruitment until the commencement of the national phase III trial, BC2001 in August 2001 (www.bc2001.org.uk), which is funded by Cancer Research UK and supported by National Cancer Research Network. The high pathological response rate contrasts with that reported in radiotherapy series and suggests a significant radiosensitising effect. The response rate is consistent with that seen in previous chemoradiotherapy series and in particular is consistent with the chemoradiotherapy arm of the randomised study reported by Coppin *et al* (1996). Furthermore, the response rate quoted in that study for radiotherapy alone was 52%. In addition, in our group, a number of patients had macroscopically normal bladders at follow-up but biopsy confirmed microscopic persistent disease. Thus the usual cystoscopic follow-up with biopsy reserved for patients with visible disease may overestimate reported response rates. Patients receiving this regimen had particularly high rates of local control with a 1-year local progression free rate of 82 and 79% at 2 years. It is worth mentioning that in our series, 20 out of 41 patients presented with hydronephrosis, a poor prognostic factor for patients with bladder cancer and 17 out of 41 patients had a GFR <50 ml min^−1^, which would have excluded them from a cisplatin-containing regimen. Additionally, in this study population, 76% of patients had T3 or T4 disease and 76% had poorly differentiated (G3) disease. Overall, 12-month survival was 68%, 24-month survival was 49% and 60-month survival was 36%. A number of patients (seven out of 24) developed metastatic disease in the absence of local failure – of 19 deaths, 12 patients were still disease free in the bladder. This suggests that the reason for treatment failure was the presence of occult metastatic disease at the time of treatment, rather than failure to control the primary tumour leading to local and distant failure. This highlights the need for more effective systemic therapies for the disease in addition to methods of improving local control. The US Intergroup study (Grossman *et al*, 2003) and a large meta-analysis (Advanced Bladder Cancer Meta-analysis Collaboration, 2003) have both recently reported positive results with the use of neo-adjuvant chemotherapy, which should be regarded as the standard of care. Most treatment-related effects settled down over the weeks after completion of therapy. At the median follow-up of 50.7 months, we have not seen any late bowel or bladder toxicity. Five patients proceeded to salvage cystectomy, three for persistent but downstaged disease (one from T3 to T1, and two from T2 to CIS) and two for recurrent disease. There was no evidence that the addition of chemotherapy to radiotherapy had compromised subsequent surgery, although clearly numbers are too small to draw definite conclusions. Patients with persistent muscle invasive disease did not undergo cystectomy secondary to other comorbid conditions that precluded them from the option of salvage cystectomy. All the patients who were fit for surgery at the time of recurrence were offered the option of salvage cystectomy.

Overall, this regimen has encouraging activity with acceptable toxicity in a poor prognosis group of patients many of whom would not have been fit for platinum-based chemotherapy. The schedule can be given as an outpatient in most cases. Response rates are comparable to those reported in previous chemoradiotherapy series, including the one randomised comparison with radiotherapy. The outcomes for large locally advanced bladder tumours with extravesical extension are poor regardless of the treatment modality used (Bassi *et al*, 1999; Gschwend *et al*, 2002). A majority of patients with complete pathological response retain their bladders free from invasive relapse, while about one-quarter will develop superficial recurrence. This may be managed in the standard fashion with transurethral resection of the bladder tumour and intravesical therapies (Zietman *et al*, 2001; Rödel *et al*, 2002b). The national randomised phase III trial, BC2001 launched in August 2001, will further define the role of synchronous chemoradiotherapy in muscle invasive bladder cancer.
